# Peptidoglycan Glycosyltransferase Substrate Mimics as Templates for the Design of New Antibacterial Drugs

**DOI:** 10.3389/fimmu.2013.00078

**Published:** 2013-03-27

**Authors:** Adeline Derouaux, Eric Sauvage, Mohammed Terrak

**Affiliations:** ^1^Centre d’Ingénierie des Protéines, University of LiègeLiège, Belgium

**Keywords:** peptidoglycan, glycosyltransferase, moenomycin, lipid II, antibacterial

## Abstract

Peptidoglycan (PG) is an essential net-like macromolecule that surrounds bacteria, gives them their shape, and protects them against their own high osmotic pressure. PG synthesis inhibition leads to bacterial cell lysis, making it an important target for many antibiotics. The final two reactions in PG synthesis are performed by penicillin-binding proteins (PBPs). Their glycosyltransferase (GT) activity uses the lipid II precursor to synthesize glycan chains and their transpeptidase (TP) activity catalyzes the cross-linking of two glycan chains via the peptide side chains. Inhibition of either of these two reactions leads to bacterial cell death. β-lactam antibiotics target the transpeptidation reaction while antibiotic therapy based on inhibition of the GTs remains to be developed. Ongoing research is trying to fill this gap by studying the interactions of GTs with inhibitors and substrate mimics and utilizing the latter as templates for the design of new antibiotics. In this review we present an updated overview on the GTs and describe the structure-activity relationship of recently developed synthetic ligands.

## Peptidoglycan Biosynthesis

The emergence of multidrug-resistant bacteria causes major public health problems, particularly in medical facilities where the lack of efficient antibiotics could jeopardize the advances made in the treatment of many diseases. New efficient antibiotics against multidrug-resistant strains are urgently needed to counter this worrying situation.

**Figure 1:**
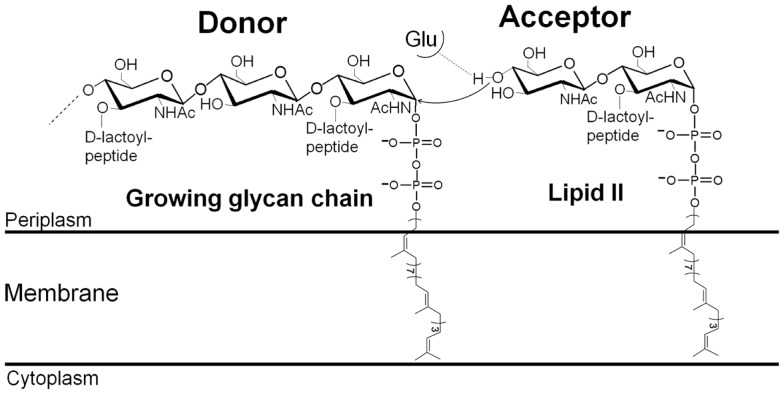
**Mechanism of glycan chain polymerization by a glycosyltransferase**.

Peptidoglycan (PG) is an essential net-like macromolecule that surrounds most bacteria, gives them their shape, and protects them against their own high osmotic pressure (Vollmer et al., 2008). PG is assembled by the membrane-bound PG synthases [glycosyltransferases (GTs) and transpeptidases (TPs)]. Its degradation or the inhibition of its synthesis leads to bacterial lysis and cell death. This makes PG a successful target for many antibiotics such as molecules of the penicillin family, which covalently bind to the TP active sites and block PG cross-linking. As a consequence, these enzymes are known as penicillin-binding proteins (PBPs) (Sauvage et al., 2008). The immediate precursor of PG is lipid II, which consists of the disaccharide *N*-acetylglucosamine (GlcNAc) and *N*-acetylmuramic acid (MurNAc)-peptide attached to the membrane-bound undecaprenyl lipid carrier via a pyrophosphate group (Figure 1). Lipid II is synthesized inside the cell by a series of cytoplasmic and membrane-associated proteins (Barreteau et al., 2008). It is flipped across the cytoplasmic membrane by the flippases of the SEDS family such as RodA, FtsW, and SpoVE involved in shape, elongation, division, and sporulation (Mohammadi et al., 2011). Once in the membrane outer leaflet, the disaccharide peptide moiety of lipid II is polymerized by the GTs, forming the nascent PG which is immediately incorporated into the preexisting PG layer by the TPs via the peptide stems (Vollmer et al., 2008).

A single bacterial cell contains several PBPs, among which are bifunctional GT/TP class A PBPs and monofunctional TP class B PBPs. Monofunctional GTs also exist in a few bacterial species (Sauvage et al., 2008). Despite their apparent redundancy PBPs are highly specialized proteins and their *in vivo* function is dictated by the morphogenetic network of partner proteins within which they operate rather than by the reaction they catalyze (den Blaauwen et al., 2008). As occurs with the inhibition of the PBP TP activity by β-lactam antibiotics, GT inhibition will block the PG synthetic machinery and lead to bacterial cell lysis.

## The Glycosyltransferases: Substrates, Structure, and Mechanism

The essential function of GTs is conserved among most bacterial species and has no eukaryotic counterparts. These characteristics and GTs’ localization on the outer face of the cytoplasmic membrane make them attractive and easily accessible targets for antibacterial compounds.

In the last decade our understanding of the GT reaction (Figure 1) at the enzymatic and structural levels has improved significantly. The characterization of several GT enzymes has been made possible thanks to the development of methods for the synthesis or isolation of labeled (fluorescent or radioactive) lipid II and lipid IV (lipid-linked tetrasaccharide) substrates and analogs (Schwartz et al., 2001; VanNieuwenhze et al., 2001, 2002; Ye et al., 2001; Breukink et al., 2003; Zhang et al., 2007; Gampe et al., 2011; Shih et al., 2011). This has allowed the development of assays to estimate their activity, such as the continuous fluorescent assay (Schwartz et al., 2002), which could be adapted to a microtiter plate format to screen for assay conditions and GT inhibitors (Offant et al., 2010; Derouaux et al., 2011). Methods for the synthesis of lipid IV substrate and higher oligosaccharides of MurNAc-GlcNAc have been described recently (Zhang et al., 2007; Gampe et al., 2011; Shih et al., 2011). They have provided a better understanding of the interaction between the GTs and their natural substrates and will facilitate the study of inhibitor specificity as well as the design of substrate-based inhibitors of the GTs.

Moenomycin is a specific inhibitor of the GTs and has been used to study their properties (Welzel, 2005). Its complete synthesis has been achieved; the synthetic genes have been identified and expressed in heterologous strains to produce defined fragments of the molecule (Taylor et al., 2006; Ostash et al., 2007). Several analogs have been prepared and characterized, including fluorescently labeled moenomycin, which was used to develop a high-throughput screening assay (Welzel, 2005; Adachi et al., 2006; Cheng et al., 2008).

**Figure 2:**
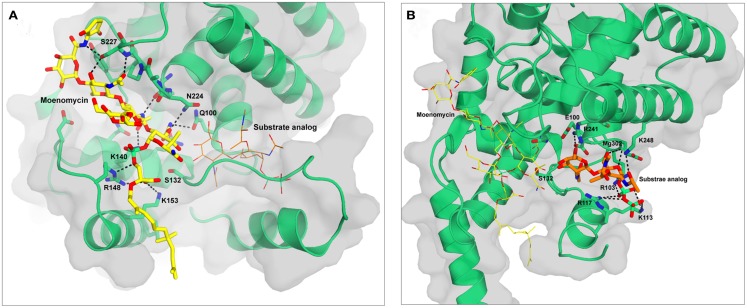
**Structures of *S. aureus* MtgA with moenomycin (A) and substrate analog 3 (B) bound in the donor and acceptor sites respectively**. The figures are based on the pdb files 3HZS **(A)** and 3VMT **(B)**. Moenomycin is shown in yellow and the substrate analog in orange. Hydrogen bonds are shown as black dotted lines. The backbone atoms of residues 223–227 are shown as sticks to highlight the hydrogen bonds they form with moenomycin.

Efforts in protein expression and purification from different bacterial species have allowed the determination of the crystal structures of GTs both in the apo form and in complex with moenomycin or analogs (Lovering et al., 2007; Yuan et al., 2007; Heaslet et al., 2009; Sung et al., 2009). The sequences and structures of the GTs are highly conserved. They all have five conserved motifs in their sequences, containing residues important for the interaction with the substrates and for the activity of the enzyme (Terrak et al., 2008). The 3D structures show that the proteins from different species have similar folds composed mainly of α-helices and similar binding modes to moenomycin with only a few variations. The GT domain is divided into a head subdomain with similarities to the phage λ lysozyme, a hydrolytic enzyme that catalyzes the breakdown of the PG polymer, and a so-called jaw domain, specific to the GT51 family. The jaw domain contains a hydrophobic region partly embedded in the cytoplasmic membrane that facilitates access to the membrane-bound lipid II substrate. The interface between these two subdomains harbors the enzymatic cavity and can be divided into two substrate binding sites: a donor site for the lipid-bound growing glycan chain and an acceptor site for lipid II (Figure 2). They are separated by a mobile region, which has only been well defined in the *Staphylococcus aureus* PBP2 and MtgA (monofunctional GT) structures (Lovering et al., 2008; Heaslet et al., 2009), and has been proposed to play a role in the catalytic mechanism. Interestingly, *Escherichia coli* PBP1b and *S. aureus* MtgA have been crystallized with their transmembrane (TM) helix (66–96 in PBP1b) (Sung et al., 2009; Huang et al., 2012). The structure of PBP1b shows that the TM residues 83–88 interact with residues 292–296 of the GT domain. Although no interaction between moenomycin and the TM segment has been observed in the crystal structure of the complex, moenomycin has been found to bind with higher affinity to the full-length *E. coli* PBP1b than to the protein without the TM segment (Cheng et al., 2008). The full-length PBP1b and MtgA with TM segments showed higher activity than the truncated forms (Sung et al., 2009; Huang et al., 2012) and the TM of *Streptococcus pneumoniae* PBP2a was found to influence the glycan chain length (Helassa et al., 2012). These reports suggest that the TM may play a role in substrate and moenomycin binding and in the GT reaction.

The direction of the elongation of the glycan chain by the GTs has been a matter of debate for a long time. It is now established that this occurs by the addition of disaccharide subunits to the anomeric (reducing) end of the growing chain (Fraipont et al., 2006; Perlstein et al., 2007) (Figure 1). The reaction catalyzed by the GTs starts with the deprotonation of the GlcNAc 4-OH of lipid II with the active site glutamate of motif 1 acting as the catalytic base (Terrak et al., 1999; Schwartz et al., 2002). The activated nucleophile then directly attacks the lipid-linked MurNAc carbon C1 of the growing polysaccharide chain leading to the formation of a new β-1,4 glycosidic bond and the elongation of the glycan chain. The GT reaction is complex because the length of the substrate in the donor site increases by two sugar residues after each catalytic cycle. *In vitro*, the processive polymerization of the glycan chain is preceded by a limiting phase of initiation, probably because lipid II is not the optimal substrate in the donor site. Using longer oligosaccharide substrates suppresses this initial lag time (Schwartz et al., 2002; Wang et al., 2011). Subsequently, chain elongation occurs in a processive way after each cycle, the lipid-bound glycan chain product must move to the donor site, probably thanks to the higher number of interactions that surround the pyrophosphate group and the sugar moieties in the donor site than in the acceptor site (Huang et al., 2012). The mechanism by which the product moves from the acceptor to the donor site, and the factors that determine the glycan chain length and control its termination remain to be determined.

## Glycosyltransferase Inhibitors

Moenomycin is the only natural product known to directly target the GT active site. In the following sections we will present the recent advances on GT binders including moenomycin derivatives and substrate mimics as GT inhibitors.

## Moenomycin and Derivatives

**Figure 3:**
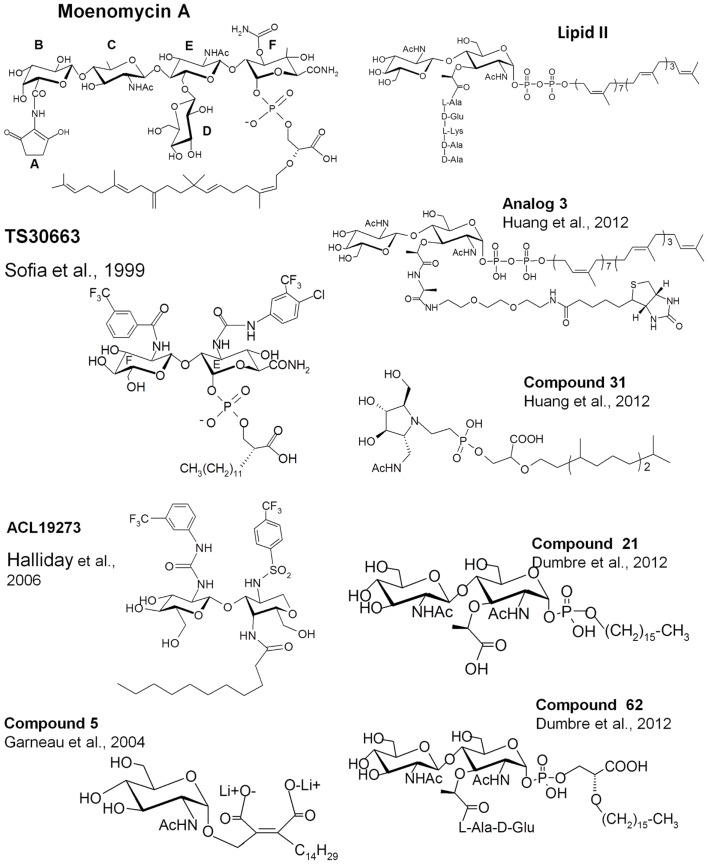
**Structures of lipid II, moenomycin, and their analogs**. The names of the compounds are as in the original papers and are indicated with the references.

Moenomycin is a natural product isolated from *Streptomyces ghanaensis* or *bambergiensis* (Welzel, 2005). Its structure consists of a pentasaccharide and a chromophore group linked to a phosphoglycerate-lipid (Figure 3). This structure is reminiscent of that of the undecaprenyl-linked growing chain (lipid IV) of PG, the donor substrate. Moenomycin is one of the most active antibiotics (MIC of 0.05 μg/ml for *S. aureus*) against a variety of Gram-positive bacteria (10–1000 times better than vancomycin) and inhibits all the GTs tested at low concentration. Interestingly, no resistance has been observed, even after moenomycin was used extensively in animal feed (Butaye et al., 2003). Moenomycin is not used clinically for the treatment of bacterial infection because of adverse properties, mainly related to its long lipid tail (Goldman and Gange, 2000). Since its discovery extensive research has been performed as it offers a good starting point for the synthesis of new molecules with better properties. All the available crystal structures of GTs in complex with moenomycin reveal a similar conformation of the moenomycin in the donor site (Figure 2A). The sugar part binds in an extended conformation and interacts with the GT via many hydrogen bonds with backbone or side chain atoms. The phosphoglycerate group of moenomycin lies in a positively charged pocket that probably accommodates the pyrophosphate group of the growing glycan chain. The two negatively charged phosphoryl and a carboxylate groups make critical contacts with conserved residues in this pocket. These interactions orient the lipid chain toward the hydrophobic groove facing the cytoplasmic membrane. The lipid group is generally not seen in the crystal structures.

Structure-activity relationship studies, reviewed by Welzel (2005), using degradation products of moenomycin or chemically synthesized fragments showed that the trisaccharide (CEF) and disaccharide (EF) degradation products inhibit the GT reaction *in vitro*, but that only the trisaccharide has an antibacterial activity. Decreasing the length of the lipid chain leads to a gradual decrease until a complete loss of antibacterial activity is observed. A structure of a GT in complex with a neryl-moenomycin has been solved, showing that this compound is still able to bind to the GT active site and to inhibit the GT *in vitro* but it lacks antibacterial activity (Yuan et al., 2008). Most of the interactions in the carbohydrate part of moenomycin are mediated by the disaccharide units EF. The carbamoyl group at C3 of the F-ring and the *N*-acetyl group at C2 of the E-ring are critical for binding. The C2-*N*-acetyl group of the C-ring also participates in binding and when the C-ring was replaced by the D-ring, the DEF compound had an antibacterial activity close to that of the CEF fragment (Yuan et al., 2008), showing that an additional and non-specific bond is sufficient to obtain antibacterial activity based on EF and a phosphoglycerate-lipid chain as the core pharmacophores (Welzel, 2005).

On the basis of this knowledge, different groups have tried to synthesize new analogs with antibacterial activity based on the EF-phosphoglycerate-lipid fragment of moenomycin (Sofia et al., 1999; Baizman et al., 2000). Sofia et al. (1999) synthesized a library of disaccharide analogs to explore modifications at C2 of the E-ring, at C3 of F-ring, and in the glycerate-lipid part. Some compounds were 10- to 20-fold more active than the disaccharide derivatives of moenomycin against Gram-positive bacteria and also showed cell wall inhibition activity (TS30663, Figure 3). They contained aromatic groups attached to the E and F rings and a shorter lipid chain of 12 carbons instead of 25. It seems that the derivatization of the E and F units creates new interactions with the enzyme active site that increase the affinity of the compounds and probably reduce the effect of the shortening of the lipid chain. These modifications generated compounds with properties different from those of the parent molecule and which may have different modes of action. Using a similar strategy, a diverse library of compounds has been generated by Alchemia Limited based on the disaccharide scaffold and where the phosphate group was replaced by an amide group to increase the stability of the molecules (ACL19273, Figure 3) (Halliday et al., 2006). A series of promising active hits have been found but, to the best of our knowledge, they have not yet reached the clinical trial stage.

## Substrate Analogs

Besides moenomycin, which competes for the donor site of the GTs with the elongating glycan chain, the only known ligand that binds to the acceptor site of the GTs is the lipid II substrate. Attempts have been made to synthesize GT inhibitors based on monosaccharide and disaccharide analogs of lipid II, some of them also exhibiting some moenomycin-like features (Hecker et al., 1990; Qiao and Vederas, 1993; Garneau et al., 2004). Most of the compounds were not active or were modest inhibitors of the GTs. A monosaccharide analog (compound 5, Figure 3), in which the pyrophosphate was replaced by a dicarboxylate, exhibited 28% inhibition of the GTs at 100 μM (Garneau et al., 2004). More recently, thanks to the availability of structural details and a better understanding of the GT reaction, reports have been published describing the synthesis and evaluation of new substrate analog inhibitors of the GTs. We have studied a series of substrate analogs (monosaccharides and disaccharides) with variations in the lipid, the pyrophosphate, and the peptide moieties (Dumbre et al., 2012). The peptide moiety contained 0, 2, or 3 residues of the sequence l-Ala-γ-d-Glu-l-Lys and the lipid part was a linear 16-carbon chain connected to the MurNAc via a phosphate or phosphoglycerate group. Although these compounds had modest GT inhibitory activity (residual activities between 7 and 84% at a 500 μM concentration) they provided a comprehensive structure-activity view shedding light on the entities required to design substrate-based inhibitors of the GTs. The disaccharide analogs were more active than their cognate monosaccharides. The best compound in the phosphate series was a disaccharide devoid of a peptide moiety (compound 21 *K*_i_ = 26 μM, Figure 3). The inhibition of GT activity by the monophosphate analogs decreased as the length of the peptide increased. Phosphoglycerate compounds were 3- to 10-fold more efficient than the cognate monophosphate compounds and in this series the disaccharide dipeptide (compound 62, Figure 3) was a better inhibitor than compound 21 (*K*_i_ 17.6 vs. 26 μM) with a MIC value of 128 μg/ml against *S. aureus*. The substitution of the C16 lipid chain by geranyl completely abolished the activity of the compounds. The specificity of these compounds for the donor and acceptor sites still needs to be determined. Except for compound 62, none of the compounds had antibacterial activity but the most active ones induced bacterial cell chaining similar to the phenotype observed with moenomycin (McPherson and Popham, 2003).

Shih et al. (2010) have synthesized lipid I (lipid II without GlcNAc) and lipid II analogs where the 4-OH of the GlcNAc is replaced by fluorine so that this compound cannot act as an acceptor. These two molecules inhibited the GT activity of *E. coli* PBP1b by 13 and 42% at 100 μM. The authors also synthesized compounds that mimic the transition state during the sugar transfer process using an iminocyclitol, a pyrophosphate mimic (a phosphono methylserine), and a lipophilic moiety. As for moenomycin, the lipophilic part was essential for GT inhibition. A compound with a branched lipid was more potent than a compound with a linear one. An ether linkage between the pyrophosphate mimic and the lipophilic moieties was five times more efficient than an amide linkage. Compound 31 (Figure 3) exhibited the best inhibitory activity (>80% at 100 μM) and an MIC value of 125 μM against *S. aureus*. This compound showed better inhibition activity of the GTs than the lipid II analog (lipid II with fluorine instead of 4-OH). The latter can serve as donor substrate and may not be efficient as an inhibitor.

The same group has synthesized lipid II analogs with variable peptide moieties and evaluated their binding to the GTs (Shih et al., 2012). Their results show that the pentapeptide and the tripeptide derivatives have similar binding affinities; the derivative with an l-Ala residue has an intermediate affinity. In the absence of peptide and upon substitution of the d-lactoyl of the MurNAc by a methyl group no binding was observed. The authors concluded that the d-lactoyl-l-Ala moiety is essential for substrate binding. We have shown that lipid II analogs with d-lactoyl have an inhibitory activity on GTs indicating that the l-Ala residue of these analogs is not required for binding and that a d-lactoyl group is sufficient (Dumbre et al., 2012).

Huang et al. (2012) reported the synthesis of lipid II substrate analogs and their co-crystallization with the MtgA from *S. aureus*. In analog 3 (Figure 3), a disaccharide undecaprenyl pyrophosphate with a pentapeptide mimic, the 4-OH of GlcNAc has been inverted (GalNAc) to avoid its utilization as acceptor substrate. This analog is expected to bind to both sites but, surprisingly, the crystal structure (pdb 3VMT) of the complex with the MtgA shows the compound bound to the acceptor site only and not to the donor site (Figure 2B). This is probably due to the low binding affinity of lipid II to the donor site and suggests that a larger oligosaccharide (lipid IV) is probably needed to bind efficiently to this site. Lipid IV has been found to have a higher affinity for GT than lipid II (Shih et al., 2011). In the crystal structure the inhibitor is deeply inserted into the acceptor site, where the enzyme residues 127–131 are observed in the structure of the MtgA-moenomycin (3HZS), and this involves a large displacement of residues 111–131. The peptidic moiety of analog 3 is not seen in the electron density. The pyrophosphate is in a positively charged pocket comprising residues Arg103, Lys113, and Arg117. The electron density is unclear and could also be accounted for by the polypeptidic chain instead of the substrate analog.

## Conclusion

Glycosyltransferase is a validated antibacterial target that has not been fully explored in the past because of technical difficulties related to the intrinsic properties of the proteins and their substrates. Most of the studies on GT activity inhibition have focused on moenomycin and its derivatives. The adverse properties of moenomycin and the need for the lipid moiety to achieve full antibacterial activity have prevented its development as an antibacterial agent in humans. With the ongoing antibiotic resistance crisis, the GT step in PG synthesis has emerged as one of the major antibacterial targets for the development of new antibiotics. This interest has resulted in a major breakthrough in the last decade: crystal structures of GT and complexes with moenomycin have been elucidated, the preparation of substrates has been improved, new assays have been developed, and particularly extensive efforts in the synthesis of substrate and moenomycin analogs have been made. Attempts to screen small molecule libraries for GT inhibition have also been investigated. This dynamic will certainly contribute to our better understanding of the GT-substrate mimic interactions and the discovery of new small molecule lead compounds. The use of these ligands as templates for the design and synthesis of new molecules may deliver useful antibacterial drugs against resistant bacteria.

## Conflict of Interest Statement

The authors declare that the research was conducted in the absence of any commercial or financial relationships that could be construed as a potential conflict of interest.
